# Exercise Interventions in Children with Cancer: A Review

**DOI:** 10.1155/2011/461512

**Published:** 2011-10-27

**Authors:** Tseng-Tien Huang, Kirsten K. Ness

**Affiliations:** Department of Epidemiology and Cancer Control, St. Jude Children's Research Hospital, Memphis, TN 38105, USA

## Abstract

The purpose of this review is to summarize literature that describes the impact of exercise on health and physical function among children during and after treatment for cancer. Relevant studies were identified by entering the following search terms into Pubmed: aerobic training; resistance training; stretching; pediatric; children; AND cancer. Reference lists in retrieved manuscripts were also reviewed to identify additional trials. We include fifteen intervention trials published between 1993 and 2011 that included children younger than age 21 years with cancer diagnoses. Nine included children with an acute lymphoblastic leukemia (ALL) diagnosis, and six children with mixed cancer diagnoses. Generally, interventions tested were either in-hospital supervised exercise training or home based programs designed to promote physical activity. Early evidence from small studies indicates that the effects of exercise include increased cardiopulmonary fitness, improved muscle strength and flexibility, reduced fatigue and improved physical function. Generalizations to the entire childhood cancer and childhood cancer survivor populations are difficult as most of the work has been done in children during treatment for and among survivors of ALL. Additional randomized studies are needed to confirm these benefits in larger populations of children with ALL, and in populations with cancer diagnoses other than ALL.

## 1. Introduction

Progress in treatments for childhood cancer have greatly improved cure rates, with 5-year survival now approaching 80% [1]. This has resulted in a growing population of childhood cancer survivors. In 2006, there were more than 11 million cancer survivors in the United States; three times the number of survivors in 1971 [2]. It is estimated that 1 in 810 individuals under the age of 20 is a survivor of childhood cancer and that 1 in 640 individuals between the ages of 20 and 39 years has successfully survived childhood cancer [3]. Improving survival rates, however, does not come without consequences. Treatment of childhood cancer is associated with a spectrum of late effects, including impaired growth and development, cognitive dysfunction, diminished neurological function, cardiopulmonary compromise, musculoskeletal sequelae, and secondary malignancy [4–6]. Oeffinger et al. [6] reported that one-third of childhood cancer survivors have severe or life-threatening medical complications 30 years after diagnosis. Therefore, attention today is focused not only on survival but also on the quality of survival. 

Impaired physical fitness has been reported during and after childhood cancer treatment [7–12]. Impaired physical fitness typically includes reduced cardiopulmonary function, decreased muscle strength, fatigue, and altered physical function. Treatments for childhood cancer, including radiotherapy, chemotherapy, and surgery, can result in acute and long-term injury to the heart, lungs, and skeletal muscles, systems necessary for optimal physical fitness [8, 13–19]. Additionally, reduced levels of physical activity both during and after treatment for childhood cancer can contribute to cardiac deconditioning and skeletal muscle atrophy, ultimately limiting opportunities for participation in recreational activities and life roles that are dependent on adequate physical fitness. Exercise intervention has the potential to improve cardiopulmonary and musculoskeletal function, perhaps preventing long-term deficits in physical fitness if incorporated during or soon after treatment in children with cancer diagnoses [20–24]. 

Another factor that may be associated with impaired physical fitness among childhood cancer survivors is cancer-related fatigue. Fatigue during and after treatment has the potential to have a negative impact on physical activity and on psychosocial well-being. A recent study reported that the prevalence of cancer-related fatigue was over three times higher in long-term survivors of childhood cancer when compared to the general population (OR: 3.29; 95% CI: 1.9–5.70) [25]. In another investigation that evaluated associations between demographic and medical factors and health-related quality of life (HRQOL) among pediatric cancer survivors, Meeske et al. [26] found that fatigue was the most powerful predictor of functional status and HRQOL. Given that there is evidence that exercise and physical activity programs can reduce fatigue, and enhance psychosocial health in survivors of adult cancer, such interventions may have a similar effect in the pediatric population [27].

This review of the literature indicates that there is growing evidence for the positive effects of physical training on organ system function, fatigue and physical well-being in children during and after treatment for cancer [20, 28]. However, the optimal intervention modality and the intensity, timing, and duration of the intervention are difficult to determine. In the published literature, very few exercise interventions undertaken in the pediatric cancer or pediatric cancer survivor populations have been randomized clinical trials, and, across studies, the components of aerobic training, resistance, and flexibility exercises are implemented with differing intensity, timing, and duration. In addition, the sample sizes are small, limited primarily to study populations with acute leukemia diagnoses, and include widely varied outcome measures, making it difficult to draw firm conclusions or compare results between trials. We summarize here the literature that describes the effects of exercise intervention on immune system function, cardiopulmonary health, skeletal muscle strength, fatigue, and overall physical well-being among children during and after treatment for cancer. 

## 2. Methods and Search Results

This paper summarizes exercise intervention studies among children with cancer and is limited to studies that tested or described exercise intervention in children diagnosed with a primary pediatric cancer when younger than 21 years of age, and, includes only manuscripts available as full-text in the English language. Studies were identified by searching the PUBMED database with the terms exercise; aerobic training; resistance training; stretching; pediatric; children; cancer. Reference lists of retrieved studies were also assessed to identify additional trials. The search of the Pubmed database initially resulted in a total of 48 citations. Of these, we excluded 31 citations (3 review only, 5 not available in English, 17 no exercise intervention, and 6 adult-cancer survivors only). We include 17 published manuscripts documenting 15 studies published by June of 2011. A review of the reference lists from the retrieved manuscripts did not identify any additional papers. When reporting the outcomes of each study, if numerical results were available, effect sizes were converted to Cohen's *d*, representing standard deviations of change or differences in standard deviations between groups [29], to allow for easier comparison of the magnitude of the exercise intervention responses among studies. 

### 2.1. Exercise Intervention Studies among Pediatric Survivors

A summary of the 15 published studies included in this review examining exercise intervention for children with cancer is shown in Tables 1 and 2. A total of 302 children with cancer, survivors of childhood cancer, or normal controls participated in the 15 trials; 46 were young adults [30] and 256 were children or adolescents [21–23, 31–43]. Of these 15 exercise interventions during or after pediatric cancer treatment, seven included a control group or control intervention, nine employed supervised training with aerobic, resistance, and/or flexibility training with or without home-based exercises [21–23, 31–40, 43], five tested enhanced physical activity (EPA) interventions [30, 34, 36, 39, 42], and one used an individualized home-based exercise program [41]. We differentiate between nonrandomized (Table 1) and randomized trials (Table 2) to highlight the need for additional experimental evidence to evaluate the effects of exercise intervention for children with cancer. Only 121 children with cancer diagnoses have participated in four randomized controlled trials; 41 were children and adolescents with ALL during maintenance chemotherapy [21, 36] and 70 were the survivors of childhood cancer with mixed diagnoses [33, 42]. We also differentiate between exercise and EPA by noting that exercise implies a specific training regimen with established frequency, intensity, and duration and that EPA includes dynamic activities completed during the performance of everyday tasks [44]. The majority of the interventions included only patients with acute lymphoblastic leukemia (ALL) diagnoses; only six studies were done in study populations with mixed diagnoses [30, 31, 34, 38, 39, 42]. One study was completed in children with ALL during the first six months of medical treatment [32], and six were completed among children with ALL during the maintenance or continuation phase of medical treatment [21, 23, 35–37, 43]. Outcome measures described included (1) immunological function [31, 35], (2) cardiovascular fitness [21–23, 35, 36, 38, 40], (3) muscle strength and flexibility [21–23, 33, 34, 36, 40, 43], (4) fatigue or sleep efficacy [30, 34, 40–42], (5) general physical function [9, 21, 32, 33, 36, 40], and (6) quality of life [9, 21, 32, 34, 39].

### 2.2. Effect of Exercise on Immune Suppression and Growth Factors

Chemotherapy treatment for pediatric cancer suppresses the immune system and may interfere with normal growth, increasing susceptibility to infection and stunting or delaying musculoskeletal development during treatment [45–47]. Concern about the effects of exercise on immune function and growth factors includes the possibility that exercise may tax an already compromised immune or endocrine system and either delay recovery or further impact normal skeletal growth [48]. A pilot study by Ladha et al. [35] investigated the effects of an acute bout (30 minutes) of exercise (heart rate 70–85% of peak oxygen uptake capacity (VO_2_ peak)) on neutrophil counts and immune function in children undergoing maintenance therapy for ALL (*n* = 4; mean age 11.3 ± 5.3 years). They found no deleterious effects of this intervention on immune function. Their work is supported by Chamorro-Vina et al. who demonstrated that a daily 3-week in-hospital moderate intensity exercise training regimen, including both supervised resistance and aerobic components, did not affect immune cell recovery in 7 children who had undergone hematopoietic stem cell transplantation [31]. This same group of authors also report no significant impact of a 3 times per week 16-week aerobic and resistance training intervention on levels of growth hormone, insulin-like growth factors, and insulin-like growth binding proteins levels (IGFBP-2 and -3) [37] in children with ALL. In this study, protein levels of IGFBPs remained stable even after 20 weeks of detraining.

### 2.3. Effect of Exercise on the Cardiopulmonary System

Cardiopulmonary fitness is impaired in children during treatment and among survivors of childhood cancer [9, 10]. Reports of the effects of exercise intervention on the cardiopulmonary system during treatment are mixed. Marchese et al. [21] examined effects of exercise on cardiovascular response in children (aged 4–15 years) receiving maintenance therapy for ALL. Participants were randomly assigned into a physical therapy (PT) intervention group with home-based aerobic training two times per week (*n* = 13) or a control (non-PT intervention) group (*n* = 15). Following a four-month intervention, these authors reported no cardiopulmonary response to training as assessed by a nine minute run-walk test. Additionally, more than 50% of the children scored below the 25th percentile for cardiopulmonary fitness when compared to the normative sample in the American Alliance of Health, Physical Education, Recreation and Dance Association Guidelines at both the pre- and posttest assessments. Similarly, Takken et al. [40] implemented a 12-week community-based exercise program in 9 children with ALL (aged 6–14 years) and found no cardiopulmonary response to training as assessed by standardized cardiopulmonary exercise testing. Of note, some children in this study complained that the training program was boring, too intense, and hard to combine with their other activities which may have limited compliance. Moyer-Mileur et al. [36], who provided a home-based intervention to children with ALL during the maintenance phase of chemotherapy, report slightly more promising results. In a much longer duration intervention, with a perhaps more palatable intensity of training, they assigned thirteen 4–10-year-old children to either 15–20 minutes of moderate to vigorous physical activity 3 times per week (*n* = 6) or to no intervention (*n* = 7). After 12 months, regular physical activity and cardiopulmonary fitness were assessed with a pedometer and a Progressive Aerobic Cardiovascular Endurance Run test (PACER) [49], respectively. The authors reported that the exercise group recorded more steps on the pedometer (*P* = 0.06, Cohen's *d* = 1.12) and performed slightly better on the PACER (*P* = 0.05, Cohen's *d* = 1.22) than the control group at the end of the intervention.

It appears that hospital type supervised exercise interventions have better cardiopulmonary outcomes than do those that are home or community based. San Juan et al. [9] reported positive results after implementation of a 16-week supervised (in-person) exercise program among 7 children with ALL, 4–7 years of age, also during the maintenance phase of chemotherapy. Their study population achieved a significant increase in both ventilatory threshold (before training 15.8 ± 3.3; after training 20.7 ± 2.9 milliliters per kilogram per minute (mL/kg/min), *P* < 0.05, Cohen's *d* = 1.58) and peak oxygen uptake (before training 24.3 ± 5.9; after training 30.2 ± 6.2 mL/kg/min, *P* < 0.05, Cohen's *d* = 0.97). A subsequent study by San Juan et al. [9] showed similar benefits for an 8-week supervised exercise training program among 8 children after HCT. 

Supervised exercise training also appears to have promise for childhood cancer survivors with long-term cardiopulmonary compromise. A study by Sharkey et al. [38] examined the effects of a 12-week aerobic training program among childhood cancer survivors who had been treated with anthracycline chemotherapy (cumulative dosage 349 ± 69 milligrams per meter squared (mg/m^2^)). Among the 10 patients who completed the twice weekly program (mean age 19 ± 3 years; mean time since diagnosis 8 ± 4 years), there was an average increase in exercise time on cardiopulmonary exercise testing (CPET) of 13 percent (%) from before to after test (*P* < 0.05, Cohen's *d* = 0.47). They also reported a trend toward improvement in peak oxygen uptake (*P* = NS, Cohen's *d* = 0.36) and anaerobic threshold (*P* = NS, Cohen's *d* = 0.58), but no significant changes in body fat, spirometer parameters, cardiac index, or stroke volume index. Unfortunately, although these 10 participants showed some improvement in exercise tolerance, their exercise capacity remained substantially lower than those of normal subjects.

### 2.4. Effect of Exercise on the Musculoskeletal System

Cancer therapy in children also impacts the musculoskeletal system. Limited range of motion, loss of muscle mass, and reduced muscle strength are common among children with cancer and among survivors [33, 50–52]. Fortunately, the early intervention research targeting these impairments is very promising. Improved muscle strength and flexibility is reported following training among children during maintenance therapy for ALL [9, 21] and in groups of children with mixed cancer diagnoses [34]. In their 12-week home-based PT intervention for children during maintenance therapy for ALL (*n* = 13 intervention group, *n* = 15 control group), Marchese et al. [21] reported that stretching and resistance training improved ankle range of motion (*P* < 0.01, Cohen's *d* = 0.62) and knee extension strength (*P* < 0.01, Cohen's *d* = 0.35). In another intervention during ALL maintenance therapy among 7 children 4–7 years of age, San Juan et al. [9] implemented resistance training for the major muscle groups and reported muscle strength gains (*P* < 0.05, Cohen's *d* = 0.85 to 1.48) after just eight weeks of training [9]. These gains were maintained after a 20-week detraining period [9]. In a longer intervention program (2 years) designed to prevent bone loss in children during treatment for ALL (intervention group *n* = 25, control group *n* = 26) Hartman et al. [33] reported that stretching and twice daily short-burst high-intensity exercise resulted in improved dorsiflexion range of motion (*P* = 0.001, Cohen's *d* = 0.94), but not in improved bone health. In a group-based physical activity intervention with a group of adolescent cancer survivors with mixed diagnoses (*n* = 10), Keats and Culos-Reed [34] reported improved upper body strength (*P* < 0.05, Cohen's *d* = 0.64) after 16 weeks of participation.

### 2.5. Effect of Exercise on Fatigue

Fatigue is a common symptom in children during and following cancer treatment [53, 54]. Both exercise and EPA type interventions show some efficacy in the management of fatigue during and after cancer chemotherapy in children [30, 34]. However, it appears that fatigue reduction also requires a training response. In three studies, where the response to training was positive, fatigue reduction was evident and even persisted, whereas in one study, where the exercise response was null, so was the fatigue reduction response. Yeh et al. [41] reported reduced levels of fatigue (*P* = 0.03, Cohen's *d* = 0.54) among children with ALL (*n* = 12) who completed a six-week home-based aerobic exercise program when compared to a control group who did not (*n* = 10), and Blaauwbroek et al. [30] reported reduced levels of fatigue (*P* < 0.005, Cohen's *d* = −0.92) and increased levels of physical activity (*P* < 0.005, Cohen's *d* = 0.94) after 10 weeks of a home-based physical activity counseling intervention in childhood cancer survivors. The Blaauwbroek study was implemented in survivors (*n* = 38) who were on average 30 years of age and 22 years from their original cancer diagnosis. Importantly, fatigue reduction was maintained in their study population at a three-year follow-up time point. The results of a study by Keats and Culos-Reed [34] also demonstrated a reduction in fatigue (*P* = 0.01, Cohen's *d* = 0.69) after a 16-week group-based physical activity intervention in survivors of pediatric cancer (*n* = 10). In contrast, a 12-week community-based exercise training program where there was no exercise response, perhaps because of noncompliance, also demonstrated no fatigue reduction response (*P* = NS, Cohen's *d* = −0.26) [40].

### 2.6. Effect of Exercise on General Physical Functioning

Suppressed immune system function, poor cardiopulmonary fitness, reduced muscle strength, and fatigue may decrease the ability of a child with cancer or a childhood cancer survivor to participate comfortably in regular physical activity. Implementation of a program of exercise or EPA, on the other hand, may improve their strength and fitness and, if it alleviates fatigue, may increase ease of movement and enable activities that have a physical component. The evidence for efficacy of exercise and EPA programs to improve overall physical functioning and mobility in survivors of pediatric cancer is mixed. Among children with ALL, four different exercise intervention studies have documented the beneficial effects of a supervised training program or home-based exercise [9, 32] on general physical functioning, whereas three other studies have failed to find a positive effect of exercise on physical functioning [21, 33, 40]. Like the impact of exercise on fatigue, the impact of exercise on physical functioning appears to require that the intervention have a training effect. San Juan et al. in their series of three manuscripts (*n* = 7) demonstrated that a supervised training program among young children with ALL or in children following HCT, consisting of both resistance and aerobic exercises, improved not only muscle strength and cardiopulmonary fitness but also functional mobility as assessed by performance on three and ten meter timed up and go (TUG) tests (Cohen's *d* −0.63 to −1.53, *P* < 0.05) [9]. Gohar et al. [32] reported improved gross motor function in nine children after implementing individualized home-based exercise programs during the early phases of treatment for ALL in nine children. However, the twelve-week supervised community-based intervention by Takken et al. [40] among 9 children during the maintenance phase of ALL treatment that had no training effect also had no impact on functional mobility. Additionally, Marchese et al. [21], who demonstrated improvements in ankle range of motion and knee extension strength, but no improvements in cardiopulmonary fitness (*P* = 0.25, Cohen's *d* = 0.57) after implementation of a 16-week home program during maintenance therapy for children (intervention group *n* = 13, control group *n* = 15) with ALL, also reported no improvements in performance on the TUG test (*P* = 0.17, Cohen's *d* = −0.55). 

### 2.7. Effect of Exercise on Health-Related Quality of Life

Six of the studies we reviewed reported a health-related quality of life outcome (HRQOL) in response to exercise training or EPA [9, 21, 32, 34, 39]. Four reported a positive effect and two no effect. Positive effects were found in three studies with no control population, making it difficult to attribute the outcomes to the intervention rather than to developmental maturation or disease recovery. Gohar et al. [32] and Speyer et al. [39] both report overall improvement in HRQOL in response (*P* < 0.001, Cohen's *d* = 1.43 to 2.32) to an individualized home-based exercise intervention [32] or to an in-hospital adapted physical activity intervention [39] among children during acute phases of treatment. Interestingly, the study by Gohar et al. [32] reported an initial reduction in HRQOL when chemotherapy was intensified during treatment among 9 children with ALL. San Juan et al. [9] also reported improved HRQOL (*P* < 0.05, Cohen's *d* = 1.1) in response to their 8-week long supervised exercise intervention among 8 children following HCT, and Keats and Culos-Reed [34], in a group of 10 adolescent cancer survivors with mixed diagnoses, reported improved HRQOL (*P* = 0.01, Cohen's *d* = 0.34) after a sixteen-week physical activity and educational intervention. These results in the adolescent survivors of mixed diagnoses persisted for at least one year following the end of the intervention. In contrast to the results of their study among children following HCT, San Juan et al. reported no effect of exercise training on HRQOL (*P* = NS, Cohen's *d* = 0.31 to 0.58) among 7 children who received 16-week supervised exercise intervention during maintenance therapy for ALL [9]. This finding is similar to that of Marchese et al., who also reported no differences between the intervention (*n* = 13) and control (*n* = 15) groups on HRQOL in their study of the effects of a 16-week home-based PT intervention among children during maintenance therapy for childhood ALL [21]. 

## 3. Conclusion

It appears that exercise training can be safely undertaken during treatment for ALL and HCT with no major effects on the immune system and that exercise does not have a deleterious effect on growth factors during treatment for ALL. The published evidence is positive for the impact of exercise on muscle strength and flexibility and mixed for the impact of exercise intervention on cardiopulmonary fitness among children with ALL during maintenance therapy, among children following HCT, and among survivors exposed to cardiotoxic agents. Fatigue and general physical function are enhanced if the intervention generates a cardiopulmonary training effect. The evidence for the effects of exercise training on HRQOL in the childhood cancer population is mixed and difficult to disentangle from the effects of disease recovery and normal maturation. The early evidence suggests that supervised hospital training is effective, likely because compliance and training intensity are assured. Home- or community-based programs appear to be less effective. Unfortunately, supervised training is expensive and often unrealistic for families who may have to travel long distances to a center that specializes in cancer care. 

Even though early results are promising, specific limitations in the existing literature do not allow us to yet be able to state with confidence that exercise interventions offer clear benefits during or after treatment for childhood cancer. There have only been four randomized trials, sample sizes have been small, and diagnosis groups included in the trials have been very limited (mostly ALL). Intent to treat type of analysis has not always been completed, and mechanisms to characterize the effects of participant dropout have not been employed. In addition, inconsistencies in exercise type, duration, and frequency, and outcome measurement prohibit conclusions that might guide how an individual clinician might prescribe exercise in practice. 

Further research is needed. Studies designed to identify and characterize the type and intensity of exercise necessary to achieve clinically meaningful positive cardiopulmonary, musculoskeletal, symptom limiting, physical function, and quality of life outcomes in children with a variety of diagnoses are necessary. These interventions must be not only safe but also realistic and portable so that children, families, and long-term survivors can adopt and incorporate exercise and physical activity into their everyday lives when they are not near the specialized center that provides care for children with cancer. Additionally, larger well-designed randomized studies that employ strong statistical methodology and that evaluate the effects of participant dropout on the outcomes are important to see if the early results from these multiple small, mostly observational trials remain positive in larger populations of children with varied cancer diagnoses. 

## Figures and Tables

**Table 1:** Description of non-randomized exercise trials in children with cancer.

First author and year	Design	Demographics	Exercise intervention (type of training, frequency, and duration)	*Main outcomes
Sharkey, 1993 [38].	Pretest/posttest trial	*N* = 10. Mixed cancer types. 5 males. Mean age at the time of the study: 19 ± 3 yrs.	Intervention: aerobic training with home exercise twice per week (week 1-2 started with 15 minutes of warm-up, 15 minutes of exercise at 60% of HRmax and 15 minutes of cool-down, week 3–6 30 minutes of exercise at 70–80% HRmax, and week 7–12 30 minutes of aerobic exercise at 70–80% HRmax plus home exercise once per week). Duration: 12 weeks of out-patient cardiac rehabilitation.	Body fat (−), spirometry (−), peak heart rate (−), peak oxygen uptake (−), anaerobic threshold (−), peak cardiac index (−), peak stroke volume index (−), or vascular resistance (−). Exercise time (+13%).

Ladha, 2006 [35].	Nonrandomized safety assessment with both a cancer and a healthy control populations. Both did the intervention.	Cancer group: *N* = 4. Children and adolescents receiving maintenance therapy for ALL. Mean age at the time of the study: 11.3 ± 5.3 yrs. Healthy controls: *N* = 6. Mean age at the time of the study: 10.8 ± 4.6 yrs	Intervention: one session (5 minutes of warm-up, 20 minutes of moderate- to high-intensity exercise, and 5 minutes of cool-down) of intermittent run-walk on a treadmill at 70% to 85% of VO_2_ peak. Controls: age-matched healthy subjects performing the same exercise intervention. Duration: 30 minutes of acute bout of exercise	An acute bout of exercise did not elicit any significant negative effects on neutrophil count.

San Juan, 2007 [43] San Juan, 2007 [23] Ruiz, 2010 [37]	Pretest/posttest trial	*N* = 7. Children receiving maintenance therapy for ALL. 4 boys. Mean age at the time of the study: 5.1 ± 1.2 yrs.	Intervention: three weekly sessions (90–120 minutes) of supervised resistance training (bench press, shoulder press, leg extension, leg curl, leg press, abdominal crunch, lower-back extension, arm curl, elbow extension, seated row, and lateral pull-down; 8–15 repetitions) and aerobic exercise (started with 10 minutes of exercises at 50% of age-predicted HRmax and progressed to 30 minutes of continuous exercise at ≥70% HRmax by the end of the program). Duration: 16 weeks plus 20 weeks of detraining, during treatment	VO_2_ peak (+), VT (+), functional mobility (+) (TUDs, 3- and 10-meter TUG) and strength tests (+) (seated bench press, seated row, and seated leg press) from before training to after training. Only increased strength remained significant after detraining. Ankle dorsiflexion range of motion (−) or QOL (−). Levels of growth hormone (−), insulin-like growth factors (−), and insulin-like growth binding proteins (−).

Keats and Culos-Reed, 2008 [34].	Pretest/posttest trial.	*N* = 10. Adolescents with cancer. 2 males. Mean age at the time of the study: 16.2 ± 1.6 (range 14–18) yrs	Intervention: physical activity and educational intervention (30 minutes of educational session, 45 minutes of aerobic training, and 15 minutes of core strength and flexibility training in the first 8 weeks; a variety of noncompetitive physical activities in the final 8 weeks) Duration: 16 weeks, a group-based physical activity intervention. Attendance rate: 81.5% over 16-week intervention.	Upper body strength (+), flexibility (+), total PA (+), QOL (+), and general fatigue (+). Participants failed to maintain their postintervention PA levels at both 3- and 12-month follow-up time points.

San Juan, 2008 [22].	Pretest/posttest trial.	*N* = 8. Children after HCT for leukemia. 4 boys. Mean age at the time of the study: 10.9 ± 2.8 yrs.	Intervention: three weekly sessions (90–120 minutes) of supervised resistance training (bench press, shoulder press, leg extension, leg curl, leg press, abdominal crunch, lower-back extension, arm curl, elbow extension, seated row, and lateral pull-down; 11 repetitions) and aerobic exercise (started with 10 minutes of exercises at 50% of age-predicted HRmax and progressed to 30 minutes of continuous exercise at ≥70% HRmax by the end of the program) Duration: 8 weeks, during treatment	Muscle strength (+), VO_2_ peak (+), functional mobility (+) (TUDs, 3- and 10-meter TUG) and self-reported health status (+). BMI (−), active and passive dorsiflexion range of motion (−), VT (−), or H max (−).

Takken, 2009 [40].	Pretest/posttest trial.	*N* = 9. Children with ALL. Mean age at the time of the study: 9.3 ± 3.2 (range 6–14) yrs	Intervention: two weekly sessions (45 minutes) of supervised resistance training (sit-ups, push-ups, head and leg raises; 30-second repetition maximum and squats 60-second repetition maximum), aerobic exercise (66–77% of HRmax in first 4 weeks, 77–90% HRmax in the following 4 weeks, and ≥90% HRmax in the last 4 weeks) and a home-based exercise program (strength, flexibility, and aerobic fitness). Duration: 12 weeks, community-based exercise program	Seventy percent of trainers were satisfied with the program. BMI (−), muscle strength (−), exercise capacity (−), functional mobility (−), or fatigue levels (−).

Blaauwbroek, 2009 [30].	Pretest/posttest trial.	*N* = 38. Adult survivors of childhood cancer (mixed cancer types). 14 males. Age at diagnosis 8.1 ± 6.7 years; time since diagnosis 21.8 ± 7.1 years. Mean age at the time of the study: 29.8 ± 8.6 yrs	Intervention: enhanced physical activity (such as walking, cycling, housekeeping, and gardening) counseling. The counselor encouraged the survivors to change their lifestyle and enhance daily physical activity to meet published exercise guidelines (i.e., at least 150 minutes of moderate-to-vigorous exercise/week) and phoned the survivors at three weeks, six weeks, and nice weeks to check goals. Feedback from a pedometer. Duration: 10 weeks of counseling.	Significant improvements in fatigue and daily steps after intervention. There was a low correlation (0.12) between increase in daily steps and the decrease in fatigue.

Speyer, 2010 [39].	Cross-over, single study design.	*N* = 30. Children with cancer (hematologic malignancy: 15, solid tumors: 12, unknown: 3). 18 males. Mean age at the time of the study: 13.6 ± 2.9 yrs.	Intervention: three weekly sessions (30 minutes) of adapted physical activity (ball games, circus arts, throwing games, shooting games, racket sports, video games, and body building). Control: standard care without adapted physical activity. Duration: four periods of enhanced physical activity (cross-over).	QOL scores in physical and psychological dimensions were higher for the children who practiced than for those who did not practice adapted physical activity during hospitalization.

Chamorro-Vina, 2010 [31].	Nonrandomized controlled trial.	Intervention group: *N* = 7. Children who had undergone HCT. 5 boys. Mean age at the time of the study: 8 ± 4 yrs. Control group: *N* = 13. 9 boys. Mean age at the time of the study: 7 ± 3 yrs.	Intervention: Five weekly sessions (~50 minutes) of supervised resistance training (arm curl, elbow extension, bench press, log extension, half squat, abdominal crunch, supine bridge, and rowing; 12–15 repetitions) (stretching exercise involving all major muscle groups) and aerobic exercise (10–40 minutes of cycle ergometry at 50% to 70% of HRmax). Control: standard care. Duration: 3 weeks, during treatment.	Fitness levels (+) (half squat) or body mass (+). Exercise intervention during inpatient stay for HCT did not affect immune cell recovery in young children with high-risk cancer.

Yeh, 2011 [41].	Nonrandomized controlled trial.	Intervention group: *N* = 12. Children and adolescents with ALL. 6 boys. Mean age at the time of the study: 11 ± 4 yrs.Control group: *N* = 10. 6 boys. Mean age at the time of the study: 12.5 ± 4 yrs.	Intervention: three weekly sessions (30 minutes) of individualized home-based aerobic exercise program (exercise intensity: 40%–60% of HRR) Control: standard care Duration: 6 weeks, during treatment	General fatigue (+). Sleep/rest and cognitive fatigue scores (−).

Gohar, 2011 [32].	Pretest/posttest trial.	*N* = 9. Children with ALL. 3 males. Median age at time of the study: 4 (range 2–14) yrs	Intervention: individualized home-based exercise program (stretching exercise: ankle dorsiflexion; 5 days/week, strengthening exercise: lower- and upper-extremity exercise; 10 repetitions 5 days/week, and aerobic exercise: walking, bike riding, and dancing 10–30 minutes; 5 days/week). Duration: 6-7 months, during treatment.	Gross motor function (+) and QOL measures (+) throughout the study (at diagnosis, induction, consolidation, interim maintenance, and delayed intensification). However, QOL scores decreased from interim maintenance to delayed intensification. The parents reported being satisfied with the PT program.

*(+) to indicate a significant effect; (−) to indicate no significant effect/change.

ALL: acute lymphoblastic leukemia; AML: acute myeloid leukemia; BMD: bone mineral density; BMI: body mass index; CNS: central nervous system; HRR: heart rate reserve; HCT: hematopoietic stem cell transplant; PA: physical activity; PT: physical therapy; QOL: quality of life; VO_2_ peak: peak aerobic fitness; HRmax: maximum of heart rate; TUDs: time up and down stair test; TUG: timed up and go test; VT: ventilatory threshold.

**Table 2:** Description of the randomized exercise trials in children with cancer.

First author and year	Demographics	Exercise intervention (type of training, frequency, and duration)	*Main outcomes
Marchese, 2004 [21].	Intervention group: *N* = 13. ALL receiving maintenance therapy. 8 males. Mean age at the time of the study: 7.6 (range, 4.3–10.6) yrs. Control group: *N* = 15. 12 males. Mean age at the time of the study: 8.6 (range 5.1–15.8) yrs	Intervention: five sessions (20 to 60 minutes immediately after initial testing, and 2, 4, 8, and 12 weeks later) of PT (stretching and strengthening exercises, supervised) and an individualized home-based exercise program (bilateral ankle dorsiflexion stretching for 30 sec 5 days per week, bilateral lower extremity strengthening 3 sets, 3 days per week, and aerobic exercises). Control: no instructions related to physical fitness and no PT intervention. Duration: 16 weeks, during treatment.	Hemoglobin level (−), ankle dorsiflexion strength (−), TUDs (−), 9-minute walk-run (−), and QOL (−). Ankle dorsiflexion range of motion (active) and knee extension strength increased in intervention group from before to after test.

Hinds, 2007 [42].	Intervention group: *N* = 14. Children and adolescents with cancer. 9 males. Mean age at the time of the study: 13.0 (range 8.5–17.4) yrs Control group: *N* = 15. 3 males. Mean age at the time of the study: 11.9 (range 7.4–18.1) yrs	Intervention: enhanced physical activity (pedaling a stationary bike-style exerciser, 30 minutes, twice daily during brief hospitalization). Control: standard care. Duration: 2–4 days, during treatment.	Sleep efficiency (+).

Moyer-Mileur, 2009 [36].	Intervention group: *N* = 6. Children receiving maintenance therapy for ALL. 3 males. Mean age at the time of the study: 7.2 ± 0.7 yrs. Control group: *N* = 7. 4 males. Mean age at the time of the study: 5.9 ± 0.7 yrs.	Intervention: an individualized exercise program (three 15–20-minute sessions of moderate-to-vigorous activity per week) and nutritional education. Control: received standard diet recommendation and performed activity as tolerated. Duration: 12 months, enhanced physical activity program	Nutrient intake (−), height (−), weight (−), or BMI (−) between intervention and control groups. No intervention effect for upper body strength (push-up completed) or flexibility (sit and reach distance). Self-reported PA (+) and a cardiovascular fitness (+).

Hartman, 2009 [33].	Intervention group: *N* = 25. Children with ALL. 14 boys. Median age at the time of the study: 5.3 (range 1.3–15.6) yrs. Control group: *N* = 26. 16 boys. Median age at the time of the study: 6.2 (range 1.7–17.1) yrs.	Intervention: preventive PT program (weekly strengthening and stretching exercise and short-burst high-intensity exercise in BMD twice per week). Control: standard care. Duration: 2 years, during treatment.	Percentage of body fat (−) or less body mass (−). BMD decreased significantly in both groups between the start and end of treatment. Motor performance (−) or ankle dorsiflexion range of motion (−) between groups.

*(+) to indicate a significant effect; (−) to indicate no significant effect/change.

ALL: acute lymphoblastic leukemia; BMD: bone mineral density; BMI: body mass index; PA: physical activity; PT: physical therapy; QOL: quality of life; TUDs: time up and down stair test.
